# The health consequences of civil wars: evidence from Afghanistan

**DOI:** 10.1186/s12889-022-14720-6

**Published:** 2023-01-23

**Authors:** Mohammad Ajmal Hameed, Mohammad Mafizur Rahman, Rasheda Khanam

**Affiliations:** School of Business, Education, Law & Arts, University of Southern Queensland, Toowoomba, QLD 4350 Australia

**Keywords:** Civil wars, Causality, MVAR, GDP, Afghanistan, I11, B23, C32

## Abstract

This study examines the effects of long-run civil wars on healthcare, which is an important component of human capital development and their causality nexus in Afghanistan using the MVAR (modified vector autoregressive) approach and the Granger non-causality model covering data period 2002Q3-2020Q4. The primary results support a significant long-run relationship between variables, while the results of the MVAR model indicate the per capita cost of war, per capita GDP, and age dependency ratio have significantly positive impacts on per capita health expenditures, whereas child mortality rate and crude death rate have negative impacts. The results of the Granger non-causality approach demonstrate that there is a statistically significant bidirectional causality nexus between per capita health expenditure, per capita cost of war, per capita GDP, child mortality rate, crude death rate, and age dependency ratio, while it also supports the existence of strong and significant interconnectivity and multidimensionality between per capita cost of war and per capita health expenditure, with a significantly strong feedback response from the control variables. Important policy implications sourced from the key findings are also discussed.

## Introduction

The long-run civil wars in Afghanistan have been astonishing. It devastated the economic, social, and technological infrastructure. This social upheaval resulted in significant outbreaks in both the public and private sectors. The prevalence of mortality rate has decreased by approximately 51 percent in Afghanistan, from 1,240 deaths per 100,000 live births in 2003 to 638 deaths per 100,000 live births in 2017 [1]. Although the long-run effects of war comprise wide dimensionality and it is essential to address each of them separately, the present study specifically attempts to analyze the effects of long-run civil wars on the healthcare systems of Afghanistan as the most contested battlefield worldwide. As a macro-level strand, health, education, and employment are the essential components of nurturing the human capital of a nation to bring welfare and prosperity, yet they have been proven to be vulnerable sectors that have received substantial assaults during wartime [2, 3]. Moreover, health spendings mainly aim to result in the efficient provision of health prospects, strengthening human capital to improve overall productivity, thus contributing to welfare and efficient economic performance [4]. It is therefore important to understand the pattern of healthcare spending in a conflict zone during wartime. Afghanistan’s healthcare system, which was dependent upon the international community’s financial assistance during the last two decades [5], and its rapidly deteriorating healthcare system under the Taliban have raised another alarming concern for a major portion of the population in Afghanistan [6]. War is an ominous phenomenon that not only limits people's access to health services but also destroys the health infrastructure of the war-effected country [7]. It is also well-evident that armed conflicts cause significant underfunding and redirection of the financial resources to combat war-driven expenses [7, 8], resulting in substantial barriers to the provision of sufficient healthcare services [9, 10].

The effects of civil wars on various socioeconomic indicators can be traced from the available literature (see, for instance, [11–19]), but it suggests thorough analysis to determine the effects of civil wars on various health indicators to assist intra-policy reconciliation between the resources directed by local governments and the interference of international funding agencies supporting parallel healthcare systems. Therefore, it is important to formulate three key questions, among all others. First, do long-run civil wars in Afghanistan constitute an extra cost burden to the local government other than that which has been directly covered by the United States and its alliance during wartime? Second, do protracted (prolonged) civil wars have the same devastating magnitude and effects on Afghanistan's overall public healthcare system as in other war-effected zones? Do long-run civil wars have causality relationships with other healthcare indicators, and can this causality nexus be traced out as a feedback response as well?

There are several studies analyzing the impact of armed conflicts on various healthcare indicators in different war-effected environments, such as Sudan [20–22], El Salvador [23], Nigeria [23–25], Kurdistan [26], Syria [26–28], and Iraq [29, 30]. Though an exception is given to the work of Walker [31] for an extensive review of health consequences of war on various health indicators in Afghanistan; other relevant studies [31, 32] have been descriptive by nature, which raise empirical debates on the confounded results presented by them. Despite the scarcity of sophisticated analysis of the effects of civil wars on healthcare in Afghanistan and answers to the formulated research questions, the present study takes a new step in the literature to fill the missing gaps and invites further empirical discussions on the health consequences of armed conflicts shifting from descriptive to advanced analytical approaches.

This article is a distinctive work in the existing literature quantifying the effects of long-run civil wars on public healthcare in Afghanistan, which is a true representation of a long-run battlefield in the world. To be specific, the contribution of the present study can be outlined as follows. First, it is the first of its kind in the existing literature for Afghanistan, which fills the missing gaps. Second, the authors employ sophisticated econometric models to estimate the effects of civil wars on health predictors proxied by per capita health expenditure to provide consistent and efficient results, though most recent studies relied upon descriptive analysis that might have led to perplexing conclusions. Third, to inform evidence-based conclusions, the study controls for relevant societal and macroeconomic variables and provides appropriate policy recommendations.

The remaining parts of the study are structured as follows. Literature review presents a brief theoretical background and reviews recent empirical literature assessing the effects of armed conflicts on healthcare indicators. Data describes the data, sources of data collection, variables, and key measurements. Methods presents the empirical and econometric methods used to analyze the data. Results presents the results of data analysis. Discussion provides a brief discussion. Conclusion concludes the study and offers some relevant policy recommendations.

## Literature review

The literature widely defines war as a state of armed conflict between group of people or states seeking either economic, political, or other hegemonic benefits [33] linked by aggressions of extensive duration and magnitude across a wide spectrum, resulting in a societal catastrophe [33, 34]. Moreover, the empirical literature also widely documents the various impacts of civil wars and armed conflicts on healthcare indicators. It reports the customary measurement of war effects on mortality rates, maternity rates, healthcare systems, and gender-specific health services during wartime and afterwards, though many of them to date are descriptive by analytical method and require reconfirmation of the type, scale, and magnitude of the effects of armed conflicts on healthcare indicators. For instance, Roberts et al. [35] conducted a survey to compare the mortality rate due to inefficiency of healthcare services before and after the war period in Iraq using monthly data and a cluster sampling approach—that is, 33 clusters consisting of household interviews. Examining the inefficiencies of the healthcare systems caused by civil wars, the authors found, though descriptively explained, that civil wars led by the invaders in Iraq had significantly devastated the healthcare systems and resulted in an increase in the mortality rate by 2.5 times higher than pre-war conditions. They confirm that civil wars had seriously negative effects on healthcare systems in Iraq (see also [36]).

Betsi et al. [37] attempted to quantify the impact of civil wars and armed conflicts on healthcare systems and human resources through an administered questionnaire and review of the records of the ministry of health in Côte d'Ivoire. Using descriptive statistics, the authors found that due to armed conflicts, there was a significant reduction in the number of health staff both in the private and public health sectors, which led to the collapse of the healthcare system, public health infrastructure, interruption of condom distribution, and lack of antiretrovirals. The authors also report a significant increase in the number of non-governmental organizations supporting healthcare centers and a substantial decrease in the number of private health clinics.

Devkota and Teijlingen [38] argue that, in contrast with an abundance of literature on the negative impact of armed conflicts on healthcare systems, they show an improvement in a number of healthcare indicators in Nepal during wartime from 1996–2006. The authors employed data from the Nepal Demographic and Health Survey and found that 16 out of 19 healthcare indicators have improved during wartime, suggesting that such improvements in healthcare systems are driven by both conflict and non-conflict factors in Nepal. However, their results might be confounded due to the statistical methods used by the author; they report a counter-example of the effects of war on healthcare systems. Elamein et al. [39] evaluated the effects of war on healthcare systems in Syria through a participative data method using data collected from Turkey’s healthcare centers in Syria and local Syrian health employees. Since November 2015, the datasets have been collected from the monitoring violence against healthcare alert network. Using descriptive data analysis techniques, the authors found a significant impact of armed conflicts on the healthcare indicators, implying that from November 2015 to December 2016, more than 938 people have been directly harmed in 402 incidents of violence against healthcare. That consists of 72% injuries and 28% deaths in Syria. The authors argue that since health centers have been attacked more than other public organizations, the negativity of their effects has been substantially higher in distracting the healthcare centers, thus affecting public health in Syria.

Kotsadam and Østby [40] examined the effects of armed conflicts on healthcare proxied by maternal mortality rate in thirty Sub-Saharan African countries, using combined geo-coded data on a number of different types of violent events from the Uppsala Conflict Data Program with geo-referenced survey data from the Demographic and Health Surveys and a sister-fixed effects model to analyze the data. The authors clustered the respondents aging from 12–45 years old into gender-specific categories. They found that local exposure to the intensity of armed conflict has a significantly negative impact on the mortality rate, giving rise to the risk of maternal deaths, whereas there were significant differences in the mortality rate in rural areas with an adverse report from educated areas. On the other hand, Lafta and Al-Nuaimi [41] descriptively explained the effects of long-term war, terrorism acts, and organized crimes on healthcare systems in Iraq during the last 40 years. The authors emphasize that civil wars have severe effects on healthcare systems, increasing the numbers of morbidity, injuries, disabilities, mortality rates, and mental problems.

Jawad et al. [42] examined the direct and indirect effects of armed conflicts and violence on healthcare systems using datasets from the World Development Indicators in 181 countries for the period spanning from 2002–2019 and panel data regression analysis with fixed effects estimators to analyze their data. According to their findings, armed conflict and violence are significantly linked with persistent excess maternal and child deaths across the world, as well as reductions in key measures indicating high reduction of availability to organized healthcare systems. Their findings also highlight the importance of protecting women and children from the indirect harms of conflict, such as the degradation of health systems and exacerbating economic outcomes. Furthermore, Ekzayez et al. [43] employed an observational method to test the effects of armed conflicts on the availability and accessibility of healthcare services in Syria from October 2014 to June 2017 using datasets that were routinely collected from 597,675 medical consultations and 11,396 events. The authors used panel data techniques with fixed effects estimators to analyze the data and found that bombardments have strong negative impacts on both consultations and antenatal care visits in Syria. They also found that access to healthcare services in war-affected areas in Syria was significantly limited for patients, while conflict incidents were found to negatively affect the utilization of routine health services. Table 1 provides some more insights into recent studies relevant to the context of this study.

**Table 1:** Some relevant studies

Author	Context	Method	Findings
Spiegel et al. [20]	Rwanda	Descriptive analysis	The healthcare needs of war-affected people show an expansion in morbidity and mortality. Armed conflicts impose negative effects on the provision of curative health services
Urdal and Che [44]	Global perspective from 1970–2005	Cross-sectional data analysis	Armed conflicts cause higher fertility, and maternal mortality rates. In neighboring countries, it causes lower maternal mortality rates, possibly indicating that health interventions among refugee and host populations are relatively successful
Abbara et al. [29]	Syria	Descriptive analysis	The long-term conflict has led to significant destruction of the health infrastructure and has increased both communicable and non-communicable diseases and raised morbidity and mortality rates
Levy and Sidel [15]	Iraq	Descriptive analysis	Armed conflicts seriously affect public health. It also effects on inadequate healthcare system, social breakdown, forced migration, internal displacement, and reporting biases
Namasivayam et al. [45]	Uganda	Logistic regression analysis	Armed conflicts have direct effects on the reduction of healthcare services in Uganda vis-à-vis the rest of the regions. However, skilled assistance at birth among women has been found significantly higher
Kadir et al. [46]	Global perspective	Descriptive analysis	Armed conflicts have both direct and indirect effects on the mortality, mentality, and psychology of people, with most pressure on children, while internal displacement and family separation are also evident as the long-run consequences of civil wars
Chukwuma and Ekhator-Mobayode [47]	Nigeria	Difference-in-difference analysis	Armed conflicts like the Boko Haram Insurgency (HBI) have a high effect on the maternal mortality rates in Nigeria. Furthermore, the BHI decreased the frequency of care visits, delivery at health centers, and delivery by skilled health professionals
Bendavid et al. [48]	Global perspective	Descriptive analysis	Besides, armed conflicts have negative effects on healthcare, more than 265 million women and 368 million children have been displaced both internally and across borders

Source: Authors’ collection

Meagher et al. [49] reviewed a comprehensive literature covering the ripple impact of armed conflicts and violence on a wide range of indicators, including gender-specific barriers to accessing essential healthcare services, water, sanitation, education, and some macroeconomic indicators, such as poverty rates, debt burdens, and unemployment rate. The authors employed multidisciplinary narrative reviews of the existing literature relevant to the political economy of health in conflict zones and conclude that armed conflicts and violence seriously affect healthcare and socioeconomic indicators in war-effected areas, while gender-specific effects—negative effects of war on women and children—were found to be relatively greater.

The review of existing literature clearly indicates two critical missing gaps about the analysis of the effects of civil wars and armed conflicts on public healthcare services. First, it reports that empirical studies are scarce analyzing the effects of long-run civil wars and armed conflicts on the healthcare services in Afghanistan during the war period. Second, the literature, however, covers other war-effected zones, such as Syria, Libya, Iraq, and African countries, but the results might be confounded due to the use of non-sophisticated and non-comprehensive statistical methods to analyze relevant data. Thus, these two significant missing gaps in the literature justify the present study and spark its importance to fill the gaps.

## Data

As per the availability of data, this study employs datasets containing observations from 2002Q3–2020Q4 for Afghanistan. The variables used in the study are consistent with the theoretical concept and recent studies and includes per capita health expenditure (as a proxy for public healthcare) as the dependent variable. Per capita cost of war as a proxy for long-run civil wars; per capita GDP as a proxy for per capita income; child mortality rate expressed as the number of the death of children under 5 years old; the crude death rate, and the age dependency ratio are used as explanatory variables. The dataset for the cost of war is collected from the U.S. Department of Defense Budget, whereas all other datasets come from the WDI (World Development Indicators) World Bank. Table 2 reflects more details about variables, symbols, descriptions, and sources of data, while it also highlights some important summary statistics.

**Table 2:** Variables’ description and summary statistics

Description of variables				Summary statistics				
Name	Symbol	Description	Sources	Mean	Max	Min	Std. Dev	Obs
Per capita health expenditure	PHE	PHE is expressed in constant 2015 US dollar and includes healthcare goods and services consumed during each year	WDI	45.57	69.99	15.80	17.24	73
Per cost of war	PCW	PCW is expressed in millions of US dollar	USDB	9.27	19.43	2.14	5.69	73
Per capita GDP	PGDP	PGDP is expressed in thousands of constant 2015 US dollar	WDI	487.77	587.56	330.30	96.55	73
Child mortality rate	CHM	CHM is expressed as the number of children deaths under 5 years old	WDI	4.90	6.20	3.10	1.11	73
Crude death rate	CDR	CDR is expressed as the number of deaths per 1000 population during the year	WDI	8.19	11.04	6.15	1.51	73
Age dependency ratio	AGD	AGD is expressed as a percentage of working-age population, younger than 15 and older than 64 years old	WDI	95.59	103.67	80.08	7.51	73

Source: Authors compilation

*Max* Maximum, *Min* Minimum, *Std. Dev* Standard deviation, *Obs* Number of observations, *USDB* US Department of Defense Budget FY2020

The main variable of interest, the cost of war, represents the total amount of US dollars spent by the US on military operations in Afghanistan over the period. The choice of this proxy is based on the accuracy of its data, whereas some other studies employed the number of deaths and casualties to proxy the civil war variable (see, for instance, [50–52]). Furthermore, the study controls for two additional variables, such as the child mortality rate and the crude death rate, which are proxies for the rate of death due to causes other than the consequences of Afghanistan’s civil wars. Per capita GDP is used to assess the effects of per capita income on health expenditure and, as a result, the nation’s healthcare services. From a theoretical viewpoint, per capita income is linked with the quantification of healthcare from three major aspects, such as better nutrition; enhancement of public health infrastructure; and the advancement of medical technology used to offer healthcare services [53]. Besides, age dependency ratio is also employed as a control variable. It is used to control the extra burden other than the consequences of civil wars on health expenditure in Afghanistan. A lower age dependency ratio facilitates better healthcare services, while a higher ratio of dependence indicates greater financial stress on the working population [54].

Table 2 indicates some important descriptive highlights in addition to describing the variables. It shows that during the period under study, the average per capita health expenditure is only $45.57 with a maximum of $69.99, whilst the average per capita cost of war stands at $9.27 million with a maximum of $19.43 million. On the other hand, the per capita GDP stands at an average of $487.77 with a maximum of $587.56. The child mortality rate and the crude death rate are averaged at 4.9% and 8.19%, respectively, indicating that the crude death rate is higher than the child mortality rate. More deepening insights show that due to a long-run war and a lack of basic healthcare and malnutrition, Afghanistan is the second country to have the highest crude death rate [55, 56]. Moreover, the age dependency ratio stands at an average of 95.59 which is relatively high in comparison with other economies. In real life, a major proportion of children and elderly are part of the labor force in Afghanistan, while the data indicates the dependency ratio as the composition of the population. All these preliminary insights require further statistical analysis that are discussed the Results of the present study.

## Methods

This section explains the econometric methods used to explore the impact of civil wars and other control variables on per capita health expenditure, presenting an integrated human capital dimension from health perspectives in Afghanistan. The study follows the empirical model of Grossman [57], which expresses the demand for good health vis-à-vis other relevant predictors, thereby, the civil wars as a key variable of interest in the present study. Thus, we initiate with the following function:

1 $$PHE_{t} = \phi + \varphi_{1} PCW_{t} + \varphi_{2} Y_{t} + \varphi_{3} CHM_{t} + \varphi_{4} CDR_{t} + \varphi_{5} AGD_{t} + \varepsilon_{t}$$

where *PHE*, *PCW*, *Y*, *CHM*, *CDR*, *AGD* are per capita health expenditures, per capita GDP, child mortality rate, the crude death rate, and the age dependency ratio, respectively. $\phi$ presents the intercept and $\varphi$ is the long-run coefficient. Equation (1) explicitly considers a lifetime view and is well-defined to explore the link between health-oriented decisions and the outcomes at the aggregate level. This insight is useful to understand the likely implications of civil wars and an aging population on the level of health systems and overall healthcare expenditure over the period of time [58]. The expected coefficient signs are $\varphi_{1} < 0,\,\,\varphi_{2} > 0,\,\,\varphi_{3} < 0,\,\,\varphi_{4} < 0,\,\,\,{\text{and}}\,\,\,\varphi_{5} < 0$. The estimation of Eq. (1) begins with the test of unit root, which is important to determine the integration order of the variables to avoid misspecification and fabricated results. To that end, the Augmented model of Dickey and Fuller (ADF) [59] and Phillips and Perron (PP) [60] are employed. Assuming that the variables follow a mixed order of integration and if we let the maximum integration to be $= m$, then in our case, $m = 2$. In such circumstances, common cointegration methods do not provide consistent results, while in Johansen’s [61–63] cointegration method, any I(2) series is defined as a sub-model of the basic vector autoregressive (VAR) model through two reduced rank conditions [64] using the $\Pi$ matrix comprising trace-statistics and max-eigenvalues to establish a long-run nexus between the predictors, accounting for any I(2) series in a sample of variables [61]. The optimal lag length both for unit root and Johansen’s cointegration equations are selected using the AIC (Akaike Information Criterion), SIC (Schwarz Information Criterion), and HQIC (Hanan-Quinn Information Criterion) in the unrestricted VAR model.

Instructed by the unit root results (see Table 3) by having mixed integrating order with maximum $m = 2$ series and based on an extensive empirical literature (see, for instance, [61, 62]), the study employs the Toda and Yamamoto’s [65] modified VAR model, which is an appropriate estimation method for the case of this study. The modification is built upon the augmented VAR model—that is, the $k + d_{\max }$ augmented with the optimal lags selected via information criteria plus allowing for lags to the number of variables plugged into the unrestricted VAR model expressed as:

2 $$y_{t} = \alpha + \sum\nolimits_{i = 1}^{k} {\beta_{i} y_{t - i} } + \sum\nolimits_{j = 1}^{k} {\phi_{j} x_{1t - j} } + \sum\nolimits_{m = 1}^{k} {\varphi_{m} x_{2t - m} } + u_{t} ,$$

**Table 3:** Results of stationarity test

Variables	Augmented Dickey-Fuller				Phillips-Perron			
	W/trend		Trend		W/trend		Trend	
	t-statistics	*p*-value	t-statistics	*p*-value	t-statistics	*p*-value	t-statistics	*p*-value
*At level*								
PCW	-4.41^a^	0.000	-4.38^a^	0.000	-3.84^a^	0.003	-3.91^a^	0.001
*At first difference*								
CDR	-4.13^a^	0.000	-4.85^a^	0.000	-4.01^a^	0.000	-4.38^a^	0.000
CHM	-3.99^a^	0.001	-3.81^a^	0.001	-3.70^a^	0.009	-4.19^a^	0.000
PGDP	-5.31^a^	0.000	-5.10^a^	0.000	-4.60^a^	0.000	-4.81^a^	0.000
*At second different*								
PHE	-6.24^a^	0.000	-6.44^a^	0.000	-5.32^a^	0.000	-5.14^a^	0.000
AGD	-8.26^a^	0.000	-8.21^a^	0.000	-8.01^a^	0.000	-7.94^a^	0.000

Source: Authors’ computations

*PCW* Per capita cost of war, *CDR* Crude death rate, *CHM* Child mortality rate, *PGDP* Per capita GDP, *PHE* Per capita health expenditure, *AGD* Age dependency ratio. Optimal lag length is based on SIC

^a^indicates significance at 1% level

where for brevity, $y_{t}$ presents per capita health expenditure, which is the dependent variable; $x_{1}$ is the per capita cost of war; $x_{2}$ presents the set of control variables, such as per capita GDP, child mortality rate, age dependency ratio, and crude death rate; $k$ is the number of optimal lag length; $\alpha$ is the intercept; $\beta_{i} ,\,\,\,\phi_{j} ,\,\,{\text{and}}\,\,\,\varphi_{m}$ are the short-run dynamic coefficients of the equations’ adjustment for long-run equilibrium; and $u_{t}$ is the error term. Now, we build upon Eq. (2) using the $k + d_{\max }$ approach to test the Granger non-causality null by modified Wald statistics using Toda and Yamamoto’s [65] modified VAR model as:

3 $$y_{1t} = \theta_{1} + \left( {\sum\nolimits_{i = 1}^{k} {\lambda_{1t} x_{t - 1} + } \sum\nolimits_{i = k + 1}^{{d_{\max } }} {\lambda_{2t} x_{t - 2} } } \right) + \left( {\sum\nolimits_{i = 1}^{k} {\varphi_{1t} x_{t - 1} + } \sum\nolimits_{i = k + 1}^{{d_{\max } }} {\varphi_{2t} x_{t - 2} } } \right) + \varepsilon_{t1} ,$$

4 $$x_{1t} = \theta_{2} + \left( {\sum\nolimits_{i = 1}^{k} {\eta_{1t} x_{t - 1} + } \sum\nolimits_{i = k + 1}^{{d_{\max } }} {\eta_{2t} x_{t - 2} } } \right) + \left( {\sum\nolimits_{i = 1}^{k} {\vartheta_{1t} x_{t - 1} + } \sum\nolimits_{i = k + 1}^{{d_{\max } }} {\vartheta_{2t} x_{t - 2} } } \right) + \varepsilon_{t2} ,$$

where $\theta$ is the intercept, $\lambda_{1} ,\,\lambda_{2} ,\,\varphi_{1} ,\,\,\varphi_{2} ,\,\,\eta_{1} ,\,\,\eta_{2} ,\,\vartheta_{1} ,\,\,{\text{and}}\,\,\vartheta_{2}$ present the short-run dynamic coefficients of the model, $k$ is the number lag, and $k + d_{\max }$ is the number of cointegrating vectors of the predictors augmented into the model. Equation (3) and (4) follow asymptotic chi-squared distribution using degree of freedom based on $k + d_{\max }$ to test the null of Granger non-causality between the variables [66]. The modified VAR approach of the Toda and Yamamoto [65] to Granger non-causality has several advantages over other cointegrating vector regressions. First, it produces both adjusted VAR coefficients and Granger non-causality results simultaneously by one estimation. Second, it allows I(2) series estimation, while using other regression models may produce inconsistent and inaccurate results [67]. Finally, the study computes and reports relevant diagnostic tests to ensure the statistical validity of the results upon which the conclusions are drawn.

## Results

### Stationarity results

The analysis begins with the unit root analysis using the ADF and PP methods and reports the results in Table 3. Although the results of both ADF and PP are consistent and indicate the same findings, they show that the per capita cost of war is the only variable that is stationary at level. The crude death rate, child mortality rate, and per capita GDP are strongly significant to reject the null of non-stationarity after the first difference. Moreover, the results of both the ADF and PP methods reveal that per capita health expenditure and age dependency ratio are integrated of order two, meaning that their t-statistics are insignificant to reject the null both at level and first difference, whilst the null is rejected after their second differences at a 1% level. The results show that the variables have mixed integrating orders, such as [I(0), level stationary], [I(1), first differenced stationary], and [I(2), second differenced stationary], informing the appropriate model specification discussed in the preceding section. Since the t-statistics for both trend and without trend vector models are similar, the study concludes that there are no structural breaks in the data and proceeds with further analysis.

### Cointegration results

Using Eqs. (3) and (4), that is, Johansen’s cointegration test with considering the second difference order of integration (see, for instance, [64]), we estimate the long-run relationships between per capita health expenditure, per capita cost of war, and other control variables and report the results in Table 4. The results indicate that there are four cointegration ranks among the variables, each exhibiting significant *p*-values at 1%, 5%, and 10% levels.

**Table 4:** Cointegration test results

Cointegration ranks	Eigenvalues	Trace statistics		Max statistics	
		Trace values	*p*-values	Max values	*p*-values
1	0.44	138.57^a^	0.000	80.98^a^	0.000
2	0.39	97.58^a^	0.000	68.24^a^	0.000
3	0.32	61.87^a^	0.001	55.90^a^	0.003
4	0.24	34.27^b^	0.014	40.98^b^	0.039
5	0.18	14.58^c^	0.068	27.59^c^	0.049
6	0.00	0.19	0.658	0.00	0.658

Source: Authors’ computations

^a,b,c^indicate significance at 1%, 5%, and 10%, respectively. Null hypothesis $H_{null} :r = 0$ vs. $H_{alt.} :r \ge 1$

### Toda-Yamamoto modified VAR results

For two obvious reasons, this study employs the Today-Yamamoto modified VAR model to test both the effects of variables on per capita health expenditures and their causality relationships. First, the use of other common regressions, such as standard and restricted VAR models and the ARDL method, are not appropriate for variables that follow higher degrees of integration than I(1), as in our case. Second, standard and restricted VAR models may produce spurious results due to the existence of cointegrating ranks among variables showing I(2) series. Thus, this study estimates the Toda-Yamamoto’s [65] model to overcome these empirical shortcomings and reports the results in Tables 5 and 6. The optimal lag length of two has been selected using AIC, SIC, and HQIC criteria in standard VAR environment with maximum six lags estimation. Thus, it only reports the results of lag two estimates.

**Table 5:** Modified VAR estimates

Variables	$PHE_{t - i}$	$PCW_{j - t}$	$PGDP_{m - t}$	$CHM_{n - t}$	$CDR_{v - t}$	$AGD_{u - t}$
Coefficients	16.84^b^	10.16^b^	1.99^b^	-0.74^b^	-21.33	1.58^a^
t-statistics	4.31	6.01	4.86	-8.11	-1.60	2.71
*p*-values	0.000	0.000	0.000	0.000	0.425	0.023
Diagnostic checks						
Adjusted r-squared	0.92					
Residual heteroskedasticity	2.75	[0.355]				
F-statistics serial correlation	0.47	[0.991]				
Jarque–Bera normality of residuals	1.36	[0.625]				

Source: Authors’ computations

*PHE* Per capita health expenditure, *PCW* Per capita cost of war, *PGDP* Per capita GDP, *CHM* Child mortality rate, *CDR* Crude death rate, *AGD* Age dependency ratio

^a,b^indicate significance at 5% and 10% levels, respectively

**Table 6:** Granger non-causality estimates

Causality direction	$\boldsymbol k\boldsymbol+{\boldsymbol d}_{\mathbf m\mathbf a\mathbf x}$	$\boldsymbol\chi^{\mathbf2}\boldsymbol-\boldsymbol s\boldsymbol t\boldsymbol a\boldsymbol t\boldsymbol.$	*p*-values	Causality direction	$\boldsymbol k\boldsymbol+{\boldsymbol d}_{\mathbf m\mathbf a\mathbf x}$	$\boldsymbol\chi^{\mathbf2}\boldsymbol-\boldsymbol s\boldsymbol t\boldsymbol a\boldsymbol t\boldsymbol.$	*p*-values
DV = PHE				DV = PCW			
PCW ≠ > PHE	2 + 5	28.38^a^	0.000	PHE ≠ > PCW	2 + 5	17.08^a^	0.000
PGDP ≠ > PHE	2 + 5	15.09^a^	0.000	PGDP ≠ > PCW	2 + 5	21.71^a^	0.000
CHM ≠ > PHE	2 + 5	21.45^a^	0.000	CHM ≠ > PCW	2 + 5	12.35^a^	0.000
CDR ≠ > PHE	2 + 5	16.17^a^	0.000	CDR ≠ > PCW	2 + 5	4.40	0.110
AGD ≠ > PHE	2 + 5	27.34^a^	0.000	AGD ≠ > PCW	2 + 5	3.95	0.113
DV = PGDP				DV = CHM			
PHE ≠ > PGDP	2 + 5	20.01^a^	0.000	PHE ≠ > CHM	2 + 5	14.33^a^	0.000
PCW ≠ > PGDP	2 + 5	17.14^a^	0.000	PCW ≠ > CHM	2 + 5	5.75^c^	0.71
CHM ≠ > PGDP	2 + 5	28.65^a^	0.000	PGDP ≠ > CHM	2 + 5	9.88^a^	0.007
CDR ≠ > PGDP	2 + 5	15.99^a^	0.000	CDR ≠ > CHM	2 + 5	9.51^a^	0.009
AGD ≠ > PGDP	2 + 5	18.04^a^	0.000	AGD ≠ > CHM	2 + 5	7.73^b^	0.019
DV = CDR				DV = AGD			
PHE ≠ > CDR	2 + 5	15.71^a^	0.000	PHE ≠ > AGD	2 + 5	14.02^a^	0.000
PCW ≠ > CDR	2 + 5	14.23^a^	0.000	PCW ≠ > AGD	2 + 5	3.36	0.194
PGDP ≠ > CDR	2 + 5	6.16^b^	0.046	PGDP ≠ > AGD	2 + 5	15.26^a^	0.000
CHM ≠ > CDR	2 + 5	7.10^b^	0.042	CHM ≠ > AGD	2 + 5	2.17	0.324
AGD ≠ > CDR	2 + 5	7.39^b^	0.024	CDR ≠ > AGD	2 + 5	6.87^b^	0.032

Source: Authors’ computations

^a,b,c^indicate significance at 1%, 5%, and 10% levels, respectively.$k + d_{\max }$ = optimal lag length plus lag of parameters. ≠  > indicates non-causality direction. *DV* Dependent variable of different vector model

Table 5 presents the results of the modified VAR estimates using $$k + d_{\max }$$ approach. The results are interesting and demonstrate that the per capita cost of war has a significant impact on per capita health expenditures, implying that a million US dollar increase in the per capita cost of war causes an increase in per capita health expenditure by $10.161 per quarter. It reveals that, other than those costs that have been covered by the US Department of Defense Budget for military operations, the civil war has also had its general effect on per capita health expenditure. Therefore, the incremental cost of the per capita health expenditures vis-à-vis the average per capita health expenditures as a result of the civil wars can be estimated as $$\$ 10.161/\$ 45.57 = 0.2229\,\,{\text{or}}\,\,\,22.29\%$$. Moreover, the results show that per capita GDP significantly increases per capita health expenditures by $1.99. It shows that a one hundred US dollar increase in per capita GDP contributes to increasing the per capita health expenditure by $1.99. Incorporating the child mortality rate into the model, the results show that it has negative effects on per capita health expenditures in Afghanistan. The result for the crude death rate on per capita health expenditure is insignificant, although it requires more insights into their causality nexus that come in the next section. Furthermore, the findings reveal that the age dependency ratio is positively associated with per capita health expenditure. It shows that one percent increase in age dependency ratio increase the per capita health expenditure by $1.589. Finally, the robustness of the modified VAR model is estimated and reported underneath the Table 5. They indicate that the results are statistically robust and do not suffer from heteroskedasticity and serial correlation, while their residuals are also normally distributed.

### Granger non-causality results

The key test of interest is the Granger non-causality amid predictors that is estimated using the modified VAR model of Today and Yamamoto’s [65] approach. Regardless of the empirical criticism leveled at the presentation of modified VAR estimates and the preference for the Granger non-causality results, this study reported the estimates of the modified VAR model in the preceding section and continues to present the study’s main findings extracted from the Granger non-causality test. Table 6 reports the results of the Granger non-causality estimates for all vector models estimated on the Today-Yamamoto’s modified VAR model. The reason for estimating all vector models is based on the assumption of interconnectivity among variables and the extraction of multidimensional causality between them. For the per capita health expenditure vector model, there are bidirectional causality relationships between per capita health expenditure, per capita cost of war, per capita GDP, child mortality rate, crude death rate, and the age dependency ratio, rejecting the null of Granger non-causality at 1% significant level. For the per capita cost of war vector model, there is bidirectional causality between per capita GDP, per capita cost of war, per capita health expenditure, and child mortality rate, while there is unidirectional causality running from crude death rate and age dependency ratio to per capita cost of war. For the age dependency ratio vector model, the results show that there is a bidirectional causality relationship between per capita health expenditure, per capita GDP, age dependency ratio, and crude death rate. For per capita GDP vector model, the results demonstrate that per capita GDP is bidirectionally caused by per capita cost of war, child mortality rate, age dependency ratio, and the crude death rate, supporting the rejection of null hypothesis of Granger non-causality at 1% and 5% significant levels. Moreover, the findings reveal that except for child mortality rate and age dependency ratio, all other vector models exhibit significant interdependencies and multidimensionality between the variables.

## Discussion

This study hypothesized the effects of civil wars on healthcare in Afghanistan during wartime. The statistical analysis began with the test of stationarity and revealed that the predictors follow mixed and complex integrating orders (see Table 3). This led the study to examine the long-run relationship between per capita cost of war, per capita health expenditure, and the control variables and to inform appropriate model specification. The rejected null of no cointegration ranks (see Table 4) reveals that the variables have a long-run nexus, implying that per capita health expenditure moves together with the per capita cost of war, child mortality rate, the crude death rate, per capita GDP, and the age dependency ratio in the long run. Furthermore, the results indicated that per capita cost of war, the key variable of interest, has a significantly positive impact on per capita health expenditure (see Table 5). Intuitively, the incremental effects of the civil war predictor on health expenditure do not necessarily imply an improvement in the healthcare system and its coverage, but rather an additional burden of costs being paid by the government to cover the negative health consequences of Afghanistan’s armed conflicts. Per capita GDP is also found to have positive effects on per capita health expenditure. The results are consistent with the findings of Fedeli [68], Erçelik [69], and Bayar et al. [70], who also found that an increase in GDP significantly causes the per capita health expenditures to increase over time. Moreover, the results also correspond with the findings of Rahman et al. [71], who revealed that an increase in GDP improves population’s health status in SAARC-ASEAN region. These findings reflect the fact that Afghanistan’s civil war had severe impacts on a variety of socioeconomic and healthcare indicators, including per capita health expenditures. The results lend statistical support to the findings of Mirzazada et al. [72], who found that the armed conflicts in Afghanistan have a long-run relationship with the health predictors, indicating that the increase in child mortality rate is due to several factors—an important of which is the redirection of resources to defense and military operations, rather than the advancement of healthcare services. Moreover, Malik and Akhtar [73], Hu and Mendoza [74], Jaba et al. [75], Rahman et al. [76], Ray and Linden [77], and Malik et al. [78] discovered a long-run relationship between health expenditure and child mortality rates in different geographical contexts. In contrast to our results, they found that an increase in per capita health expenditure causes the child mortality rate to decrease due to providing more healthcare services to the citizens. The findings also revealed that the age dependency ratio has a positive effect on per capita health expenditure consistent with those of Boz and Ozsarı [79], who also discovered the significance of the age dependency ratio on health expenditure. Considering the significant impact of the per capita cost of war on health expenditure, it suggests to delve into their causality directions. The Granger non-causality results shown in Table 6 highlighted two key findings. First, the results indicate that there are bidirectional causality relationships between per capita health expenditure, per capita cost of war, per capita GDP, child mortality rate, crude death rate, and age dependency ratio. Second, there exists a multidimensional and complex interdependence between the explanatory variables, indicating serious policy implications for Afghanistan when developing relevant policies. The results are consistent with those of Mehrara et al. [80], Sghari [81], Lago-Peñas et al. [82], Marta and Noelia [83], Ashiabi et al. [84], Owusu et al. [85], Lakshmana [86], Linden and Ray [87], Akif and Torusdağ [88], Lopreite and Zhu [89], Yetim et al. [34], Rahman and Alam [90], Doğan et al. [91], Dhrifi [92], Abbas and Awan [93], Bilgili et al. [94], and Yang et al. [95], who also provided statistical evidence on the significant relationships between health spending, gross domestic product, child mortality rate, and the age dependency ratio in different geographical contexts. the present study, in addition to extending the findings for Afghanistan, adds to the existing literature and provides evidence on the bidirectional causality nexus between health spending and long-run civil war proxied by the per capita cost of war in Afghanistan.

## Conclusion

Recognizing the importance of the healthcare system and its effectiveness in a war-torn society such as Afghanistan that has experienced more than four decades of consecutive civil wars, this article examines the effects of civil wars on healthcare in Afghanistan over the period from 2002Q3 to 2020Q04. To test the developed hypotheses, this study uses datasets collected from WDI (World Development Indicators) and the US Department of Defense Budget sources, and based on the time-series properties of the data—that is, the combination of I(0), I(1), and I(2) integrating orders—it employs the modified vector autoregressive (MVAR) of Today and Yamamoto [65], and expands its analysis by using the Granger non-causality method. For the rejected null of no cointegration, the results of the Johansen test confirm the existence of a significant long-run relationship between predictors. Using the MVAR technique, the results indicate that the per capita cost of war has a significantly positive impact on per capita health expenditures, which is not necessarily an indication of improvement in health systems, but rather, an additional cost burden to offset the health consequences of the civil wars. Furthermore, the findings show that per capita GDP and age dependency ratio have a significant positive impact on per capita health expenditures, whereas child mortality rate and crude death rate have a negative impact. Far more interesting results are achieved from the Granger non-causality method, which is the key test of interest in this study. It demonstrates that there is a statistically significant bidirectional causality relationship between per capita health expenditure, per capita cost of war, per capita GDP, child mortality rate, crude death rate, and age dependency ratio, while it also supports the existence of strong and significant interconnectivity and multidimensionality between civil war, per capita health expenditure, and other augmented predictors in the study. The findings have an important policy implication. It clearly indicates that civil wars create additional health expenditure without impacting the improvement of the quality of healthcare systems in Afghanistan during wartime, resulting in a significant reallocation and redirection of the healthcare budget. Thus, the government should focus on the dual effects of the civil wars and reformulate relevant policies to increase efficiency both for the advancement of the healthcare system in the long-run and the accessibility of the citizens to healthcare services, in addition to covering the proportional cost of the health consequences of the civil wars.

### Limitations of the study

Although the findings are consistent and cannot be doubted in any way; this study is confronted with one major limitation—the unavailability of the disaggregated civil war dataset by state. However, because the intensity of civil war in different states of Afghanistan has significantly varied throughout the period, this study mainly relied on aggregated civil war datasets. Future studies may account for this limitation as long as the required datasets are made available for analysis.

## Data Availability

Datasets relevant to PHE, PGDP, AGD, CDR, CHM, and population are collected from the World Development Indicators (WDI) sources available at (https://databank.worldbank.org/source/world-development-indicators) and dataset relevant to cost of war is collected from the Department of Defense Budget of the United States available at (https://www.state.gov/countries-areas/afghanistan/).

## References

[1] Zoaib A (2022). Habib Tharwani, Prince, Kumar Sean, Kaisser, Shaeen Zarmina, Islam, Mohammad Yasir, Essar, Shoaib, “Maternal mortality in Afghanistan: Challenges, efforts, and recommendations”. Clin Epidemiol Glob Heal.

[2] Tavares W (2018). Impact of Terrorist Attacks on Hospitals. J Emerg Nurs.

[3] Druetz T, Browne L, Bicaba F, Mitchell M. I, Bicaba A. “Effects of terrorist attacks on access to maternal healthcare services: A national longitudinal study in Burkina Faso,” BMJ Glob Heal. 2020;5(9). 10.1136/bmjgh-2020-002879. 10.1136/bmjgh-2020-002879 PMC7520815 32978211

[4] Qaiser Gillani D (2021). The nexus between sustainable economic development and government health expenditure in asian countries based on ecological footprint consumption. Sustain.

[5] Mirzazada S (2020). “Impact of conflict on maternal and child health service delivery: A country case study of Afghanistan. Confl. Health..

[6] Safi H (2022). Najibullah, Anwari, Palwasha, Safi, “Afghanistan’s health system under the Taliban: key challenges”. Lancet.

[7] Gesesew H (2021). The impact of war on the health system of the Tigray region in Ethiopia: An assessment. BMJ Glob Heal.

[8] Howell E, Waidmann T, Birdsall N, Holla N, Jiang K (2020). The impact of civil conflict on infant and child malnutrition, Nigeria, 2013. Matern Child Nutr.

[9] Ojeleke O, Groot W, Bonuedi I, Pavlova M (2022). The impact of armed conflicts on health-care utilization in Northern Nigeria: A difference-in-differences analysis. World Med Heal Policy.

[10] Birch M, Van Bergen L (2021). The long-term health consequences of enduring armed conflict and a lost pandemic opportunity. Med Confl Surviv.

[11] Kienzler H, Sapkota RP (2020). The Long-Term Mental Health Consequences of Torture, Loss, and Insecurity: A Qualitative Study Among Survivors of Armed Conflict in the Dang District of Nepal. Front Psychiatry.

[12] Lutz C, Mazzarino A (2020). War and Health : The Medical Consequences of the Wars in Iraq and Afghanistan. New York Univ Press.

[13] Moreno-Chaparro J, Piñeros-Ortiz S, Rodríguez-Ramírez L, Urrego-Mendoza Z, Garzón-Orjuela N, Eslava-Schmalbach J (2022). Mental health consequences of armed conflicts in adults: an overview. Actas Esp Psiquiatr.

[14] Openshaw (2012). The Health Consequences of Armed Conflict in Sub-Saharan Africa: How Much Do Conflict Intensity and Proximity Matter. African Confl Peacebuilding Rev.

[15] Levy BS, Sidel VW (2016). Documenting the Effects of Armed Conflict on Population Health. Annu Rev Public Health.

[16] Salazar MA, Law R, Winkler V (2018). Health consequences of an armed conflict in Zamboanga, Philippines using a syndromic surveillance database. Int J Environ Res Public Health.

[17] Zwi A, Ugalde A (1991). Political violence in the third world: A public health issue. Health Policy Plan.

[18] Levy BS, Sidel VW (2013). Adverse health consequences of the Iraq War. Lancet.

[19] Levy BS, Sidel VW (2011). Adverse health consequences of US Government responses to the 2001 terrorist attacks. Lancet.

[20] Spiegel PB, Checchi F, Colombo S, Paik E (2010). Health-care needs of people affected by conflict: future trends and changing frameworks. Lancet.

[21] Godfrey N, Kalache A (1989). Health needs of older adults displaced to Sudan by war and famine: Questioning current targeting practices in health relief. Soc Sci Med.

[22] Kim G, Torbay R, Lawry L (2007). Basic health, women’s health, and mental health among internally displaced persons in Nyala Province, South Darfur, Sudan. Am J Public Health.

[23] Ugalde A, Selva-Sutter E, Castillo C, Paz C, Canas S (2000). Conflict and health: The health costs of war: Can they be measured? Lessons from El Salvador. Br Med J.

[24] Dunn G (2018). The impact of the Boko Haram insurgency in Northeast Nigeria on childhood wasting: A double-difference study. Confl Health.

[25] U. E. Ekhator-Mobayode and A. Abebe Asfaw, “The child health effects of terrorism: evidence from the Boko Haram Insurgency in Nigeria,” Appl. Econ., vol. 51, pp. 624– 638, 2019, doi: 10.1080/00036846.2018.1502871.

[26] Gurses M, Ozturk AE (2020). Religion and Armed Conflict: Evidence from the Kurdish Conflict in Turkey. J Sci Study Relig.

[27] Sahloul Z (2014). Health response system for Syria: Beyond official narrative. Lancet.

[28] Heisler M, Baker E, McKay D (2015). Attacks on Health Care in Syria — Normalizing Violations of Medical Neutrality?. N Engl J Med.

[29] Abbara A, Blanchet K, Sahloul Z, Fouad F, Coutts A, Maziak W (2015). The Effect of the Conflict on Syria’s Health System and Human Resources for Health. World Health Popul.

[30] Dewachi O (2014). Changing therapeutic geographies of the Iraqi and Syrian wars. Lancet.

[31] Walker S (2010). Assessing the mental health consequences of military combat in Iraq and Afghanistan: A literature review. J Psychiatr Ment Health Nurs.

[32] Jones N. et al. “The operational mental health consequences of deployment to Iraq for UK Forces.” J R Army Med. Corps. 2008;154(2). 10.1136/jramc-154-02-05. 10.1136/jramc-154-02-05 19043988

[33] Moosa IA. The economics of war: Profiteering, militarism and imperialism. Edward Elgar Publishing Ltd. 2019. https://www.elgaronline.com/display/9781788978514.xml.

[34] Yetim B, İlgün G, Çilhoroz Y, Demirci Ş, Konca M (2021). The socioeconomic determinants of health expenditure in OECD: An examination on panel data. Int J Healthc Manag.

[35] Roberts L, Lafta R, Garfield PR, Khudhairi J, Burnham G (2004). Mortality before and after the 2003 invasion of Iraq: Cluster sample survey. Lancet.

[36] Giacaman R, Wick L, Abdul-Rahim H, Wick L (2005). The politics of childbirth in the context of conflict: Policies or de facto practices. Health Policy (New York)..

[37] Betsi NA (2006). “Effect of an armed conflict on human resources and health systems in Côte d’Ivoire: Prevention of and care for people with HIV/AIDS”, AIDS Care - Psychol. Socio-Medical Asp AIDS/HIV.

[38] Devkota B, Van Teijlingen E. Understanding effects of armed conflict on health outcomes: The case of Nepal. Confl Health. 2010;4(1). 10.1186/1752-1505-4-20. 10.1186/1752-1505-4-20 PMC3003631 21122098

[39] Elamein M (2017). Attacks against health care in Syria, 2015–16: results from a real-time reporting tool. Lancet.

[40] Kotsadam A, Østby G (2019). Armed conflict and maternal mortality: A micro-level analysis of sub-Saharan Africa, 1989–2013. Soc Sci Med.

[41] Lafta RK, Al-Nuaimi MA (2019). War or health: a four-decade armed conflict in Iraq. Med Confl Surviv.

[42] Jawad M, Hone T, Vamos EP, Cetorelli V, Millett C (2021). Implications of armed conflict for maternal and child health: A regression analysis of data from 181 countries for 2000–2019. PLoS Med.

[43] Ekzayez A, Alhaj Ahmad Y, Alhaleb H, Checchi F. The impact of armed conflict on utilisation of health services in north-west Syria: an observational study. Confl Health. 2021;25(91). 10.1186/s13031-021-00429-7. 10.1186/s13031-021-00429-7 PMC8670035 34906188

[44] Urdal H, Che CP (2013). War and Gender Inequalities in Health: The Impact of Armed Conflict on Fertility and Maternal Mortality. Int Interact.

[45] Namasivayam A, González PA, Delgado RC, Chi PC (2017). The Effect of Armed Conflict on the Utilization of Maternal Health Services in Uganda: A Population-based Study. PLoS Curr.

[46] Kadir A, Shenoda S, Goldhagen J, Pitterman S (2018). The effects of armed conflict on children. Pediatrics.

[47] Chukwuma A, Ekhator-Mobayode UE (2019). Armed conflict and maternal health care utilization: Evidence from the Boko Haram Insurgency in Nigeria. Soc Sci Med.

[48] Bendavid E (2021). The effects of armed conflict on the health of women and children. Lancet.

[49] Meagher K, Attal B, Patel P. Exploring the role of gender and women in the political economy of health in armed conflict: a narrative review. Global Health. 2021;17(88). 10.1186/s12992-021-00738-9. 10.1186/s12992-021-00738-9 PMC8334332 34348740

[50] Grobar LM, Gnanaselvam S (1993). The economic effects of the Sri Lankan civil war. Econ Dev Cult Chang.

[51] Costalli S, Moretti L, Pischedda C (2017). The economic costs of civil war: Synthetic counterfactual evidence and the effects of ethnic fractionalization. J Peace Res.

[52] Heger MP, Neumayer E (2022). Economic legacy effects of armed conflict: Insights from the civil war in Aceh, Indonesia. Confl Manag Peace Sci.

[53] Raghupathi V, Raghupathi W. Healthcare Expenditure and Economic Performance: Insights From the United States Data. Front Public Heal. 2020;8(156). 10.3389/fpubh.2020.00156. 10.3389/fpubh.2020.00156 PMC7237575 32478027

[54] Kulkarni L. Health Inputs, Health Outcomes and Public Health Expenditure: Evidence from the BRICS Countries. Int J Appl Econ. 2016;31(1)72–84. Available: http://www2.southeastern.edu/orgs/ijae/index_files/IJAE March 2016 Kulkarni - March 29 2016.pdf.

[55] Gessner BD (1994). Mortality Rates, Causes of Death, and Health Status Among Displaced and Resident Populations of Kabul, Afghanistan. JAMA J Am Med Assoc.

[56] Adegboye OA, Kotze D (2014). Causes and patterns of morbidity and mortality in Afghanistan: Joint estimation of multiple causes in the neonatal period. Can Stud Popul.

[57] Grossman M (1972). On the Concept of Health Capital and the Demand for Health. J Polit Econ.

[58] Hartwig J, Sturm JE (2018). Testing the Grossman model of medical spending determinants with macroeconomic panel data. Eur J Heal Econ.

[59] Dickey DA, Fuller WA (1979). Distribution of the Estimators for Autoregressive Time Series With a Unit Root. J Am Stat Assoc.

[60] Phillips PCB, Perron P (1988). Testing for a unit root in time series regression. Biometrika.

[61] Azimi MM (2020). Mohammad Naim, and Shafiq, “Hypothesizing directional causality between the governance indicators and economic growth: the case of Afghanistan”. Futur Bus J.

[62] Wolde-Rufael Y (2005). Energy demand and economic growth: The African experience. J Policy Model.

[63] Simionescu M, Schneider N, Gavurova B (2022). Decarbonized Energies and the Wealth of Three European Nations: A Comparative Nexus Study Using Granger and Toda-Yamamoto Approaches. Front Environ Sci.

[64] Sø. Johansen, “Likelihood analysis of the I(2) model,” Scand. J. Stat., vol. 24, no. 4, pp. 433–462, 1997, doi: 10.1111/1467-9469.00074.

[65] Toda HY, Yamamoto T (1995). Statistical inference in vector autoregressions with possibly integrated processes. J Econom.

[66] Chaido D (2017). Toda-Yamamoto Causality Test between Inflation and Nominal Interest Rates: Evidence from Three Countries of Europe. Int J Econ Financ Issues.

[67] Rjoub H, Odugbesan JA, Adebayo TS, Wong WK (2021). Investigating the causal relationships among carbon emissions, economic growth, and life expectancy in turkey: Evidence from time and frequency domain causality techniques. Sustain.

[68] Fedeli S (2015). The Impact of GDP on Health Care Expenditure: The Case of Italy (1982–2009). Soc Indic Res.

[69] Erçelik G (2018). The Relationship between Health Expenditure and Economic Growth in Turkey from 1980 to 2015. J Polit Econ Manag.

[70] Bayar Y, Dan Gavriletea M, Pintea MO, Sechel IC. Impact of environment, life expectancy and real gdp per capita on health expenditures: Evidence from the eu member states. Int J Environ Res Public Health. 2021;18(24)13176. 10.3390/ijerph182413176. 10.3390/ijerph182413176 PMC8702070 34948785

[71] Rahman MM (2017). Do population density, economic growth, energy use and exports adversely affect environmental quality in Asian populous countries?. Renew Sustain Energy Rev.

[72] Mirzazada S (2020). Impact of conflict on maternal and child health service delivery: A country case study of Afghanistan. Confl Health.

[73] Malik MA, Akhtar SN (2020). Sanitation and Diarrheal Morbidity: Evidence from Afghanistan. Asian J Heal Sci.

[74] Hu B, Mendoza RU (2013). Public Health Spending, Governance and Child Health Outcomes: Revisiting the Links. J Hum Dev Capab.

[75] Jaba E, Balan CB, Robu I-B (2014). The Relationship between Life Expectancy at Birth and Health Expenditures Estimated by a Cross-country and Time-series Analysis. Procedia Econ Financ.

[76] Rahman K (2022). Mohammad Mafizur and Alam, “CO2 emissions in Asia-Pacific region: do energy use, economic growth, financial development and international trade have detrimental effects?”. Sustainability.

[77] Ray D, Linden M (2020). Health expenditure, longevity, and child mortality: dynamic panel data approach with global data. Int J Heal Econ Manag.

[78] Malik MA, Akhtar SN, Albsoul RA, Alshyyab MA. Conflict driven displacement and child health: Evidence based on mother’s nationality from Jordan Population and Family Health Survey. PLoS One. 2021:1–17. 10.1371/journal.pone.0257080. 10.1371/journal.pone.0257080 PMC8423276 34492080

[79] Boz C, Ozsarı SH (2020). The causes of aging and relationship between aging and health expenditure: An econometric causality analysis for Turkey. Int J Health Plann Manage.

[80] Mehrara M, Musai M, Amiri H (2010). The relationship between health expenditure and GDP in oecd countries using PSTR. Eur J Econ Financ Adm Sci.

[81] Sghari MBA (2013). Relationship between Health Expenditure and GDP in Developed Countries. IOSR J Pharm.

[82] Lago-Peñas S, Cantarero-Prieto D, Blázquez-Fernández C (2013). On the relationship between GDP and health care expenditure: A new look. Econ Model.

[83] Marta P, Noelia G-P (2014). About the relationship between health expenditure and GDP: more evidence. African J Bus Manag.

[84] Ashiabi N, Nketiah-Amponsah E, Senadza B (2016). The effect of health expenditure on selected maternal and child health outcomes in Sub-Saharan Africa. Int J Soc Econ.

[85] Owusu PA, Sarkodie SA, Pedersen PA (2021). Relationship between mortality and health care expenditure: Sustainable assessment of health care system. PLoS ONE.

[86] Lakshmana CM (2013). Study on Age Structure Transition and Health Expenditure in Southern States of India. J Health Manag.

[87] Linden M, Ray D (2017). Life expectancy effects of public and private health expenditures in OECD countries 1970–2012: Panel time series approach. Econ Anal Policy.

[88] Akif Arvas AM, Asst MustafaTorusdağ R. The Link Between Health Care Expenditure and Life Expectancy: Turkey (1975–2015). Int J Humanit Soc Sci Invent. ISSN (Online. 2017;6(1)2319–7722.

[89] Lopreite M, Zhu Z (2020). The effects of ageing population on health expenditure and economic growth in China: A Bayesian-VAR approach. Soc Sci Med.

[90] Rahman K (2022). Mohammad Mafizur and Alam, “Impact of industrialization and non-renewable energy on environmental pollution in Australia: Do renewable energy and financial development play a mitigating role?”. Renew Energy.

[91] Doğan İ, Tülüce NS, Doğan A (2014). Dynamics of Health Expenditures in OECD Countries: Panel ARDL Approach. Theor Econ Lett.

[92] Dhrifi A (2018). Health-care expenditures, economic growth and infant mortality: Evidence from developed and developing countries. Cepal Rev.

[93] Abbas F, Awan HS. What Determines Health Status of Population in Pakistan? Soc Indic Res. 2018;139(1). 10.1007/s11205-017-1702-5.

[94] Bilgili F, Kuşkaya S, Khan M, Awan A, Türker O (2021). The roles of economic growth and health expenditure on CO2 emissions in selected Asian countries: a quantile regression model approach. Environ Sci Pollut Res.

[95] Yang Y, Zheng R, Zhao L (2021). Population aging, health investment and economic growth: Based on a cross-country panel data analysis. Int J Environ Res Public Health.

